# Identical MicroRNAs Regulate Liver Protection during Anaesthetic and Ischemic Preconditioning in Rats: An animal study

**DOI:** 10.1371/journal.pone.0125866

**Published:** 2015-05-14

**Authors:** Tomonori Morita, Masashi Ishikawa, Atsuhiro Sakamoto

**Affiliations:** Department of Anesthesiology and Pain Medicine, Graduate School of Medicine, Nippon Medical School, 1-1-5 Sendagi, Bunkyo-ku, Tokyo, Japan; Imperial College London, Chelsea & Westminster Hospital, UNITED KINGDOM

## Abstract

Anaesthetic preconditioning (APC) and ischemic preconditioning (IPC) ameliorate liver ischemia–reperfusion (I/R) injury and are important for regulating hepatic I/R injury. MicroRNAs (miRNAs) are short, noncoding RNA molecules of 21–23 nucleotides in length, and are currently under intensive investigation regarding their ability to regulate gene expression in a wide range of species. miRNA activity is involved in controlling a wide range of biological functions and processes. We evaluated whether APC and IPC are mediated by the same miRNAs by performing comprehensive miRNA screening experiments in a rat model of hepatic I/R injury. Twenty-one rats were randomly divided into three groups (n = 7/group): control (mock preconditioning), APC, and IPC. Control rats were subjected to 60 min of hepatic ischemia followed by 4 h of reperfusion, whereas the APC and IPC groups were preconditioned with 2% sevoflurane and hepatic ischemia for 10 min prior to ischemia-reperfusion, respectively. Liver samples were collected to measure miRNA levels after 3 h of reperfusion, and gene networks and canonical pathways were identified using Ingenuity Pathway Analysis (IPA). Blood samples were collected to measure the levels of aspartate aminotransferase (AST) and alanine aminotransferase (ALT). Although haemodynamic parameters did not vary among the groups, AST and ALT levels were significantly higher in the control group than in the APC and IPC groups. Comprehensive miRNA screening experiments revealed that most miRNAs altered in the APC group were common to those in the IPC group. IPA identified five miRNAs related to the Akt–glycogen synthase kinase-3β (GSK-3β)–cyclin D1 pathway that were significantly affected by both preconditioning strategies. The application of either APC or IPC to ameliorate hepatic I/R injury results in expression of several common miRNAs that are related to the Akt–GSK–cyclin D1 pathway.

## Introduction

Repeated brief episodes of ischemia protect various organs against subsequent prolonged ischemic insults. This phenomenon, named ‘ischemic preconditioning (IPC)’, has been described in previous studies.[1–2] Hepatocyte preconditioning can be achieved with several pharmacological agents, including volatile anaesthetics, i.e., ‘anaesthetic preconditioning (APC)’. The mechanisms by which APC and IPC protect the heart,[3] brain,[4] and liver[5–6] have been investigated extensively. In particular, previous studies have shown that activation of the Akt–glycogen synthase kinase (GSK) pathway may play a key role in APC, and cyclin D1 has been suggested to participate in organ protection following IPC.[7–10]

MicroRNAs (miRNAs) are short, noncoding RNA molecules that are 21–23 nucleotides in length, and their ability to regulate gene expression in a wide range of species is currently being investigated intensively. miRNA activity is involved in controlling a wide range of biological functions and processes, including development, differentiation, metabolism, growth, proliferation, and apoptosis.[11–12] Using comprehensive miRNA screening methodology, we previously demonstrated that anaesthesia affects miRNA expression in healthy rat livers.[13] Recent studies have revealed an important role for miRNAs and their target genes in regulating hepatic ischemia and reperfusion (I/R) injury.[14–15] Despite extensive research, the precise miRNA-mediated mechanisms involved in APC and IPC protection against ischemic injury are incompletely understood. Particularly, *in vivo* miRNA studies of the comparative effects of APC and IPC on liver I/R injury under identical experimental conditions are limited. The purpose of the present study was to evaluate whether APC and IPC are mediated via common miRNAs that regulate a common cellular signalling pathway to attenuate hepatic I/R injury in rats.

## Materials and Methods

### Ethics Statement

The experimental protocols were conducted with the approval of the Animal Research Committee at Nippon Medical School, Tokyo, Japan (Approval number: 24–214).

### Surgical preparation

Twenty-one male Wistar rats (Saitama Experimental Animals Supply Co., Ltd., Saitama, Japan) weighing 301 ± 16 g were housed at 36°C with a 12-h light–dark cycle and free access to food and water. The rats were anaesthetized with an intraperitoneal injection of pentobarbital (50 mg kg^−1^). After tracheal intubation, their lungs were mechanically ventilated with oxygen-enriched air (FiO_2_ 40%–45%) set at a tidal volume of 8–10 mL kg^−1^ and respiratory frequency of 60–70 breaths per min to maintain a partial pressure of carbon dioxide (PCO_2_) within a physiological range. All rats were placed on an electric heating pad under a warming light. The left femoral artery was cannulated to monitor mean arterial blood pressure and for sampling. Normal saline solution (3 mL h^−1^) and α-chloralose (25 mg kg^−1^ h^−1^; Wako Pure Chemical Industries, Osaka, Japan), which induces a surgical level of anaesthesia, were continuously infused via the caudal vein. All procedures were carried out under aseptic conditions.[16]

### Surgical procedures

We used a well-established rat model in which 70% of the liver is subjected to ischemia.[13, 17] In brief, a midline incision was made from the xiphoid to the pubis. The liver was exposed with retractors placed in the flank, and a clamp was attached to the xiphoid and elevated. Next, the ligamentous attachments from the liver to the diaphragm were freed. The partial liver ischemia model was used to avoid splanchnic congestion. Partial liver ischemia was induced with an atraumatic bulldog vascular clamp (MA Corporation, Chiba, Japan) for 60 min by selective clamping of the portal vein and hepatic artery supplying the left lateral and median lobes of the liver, resulting in approximately 70% liver I/R injury. At the end of the experiment, the abdominal incision was closed in two layers.

### Experimental protocols

**Fig 1:**
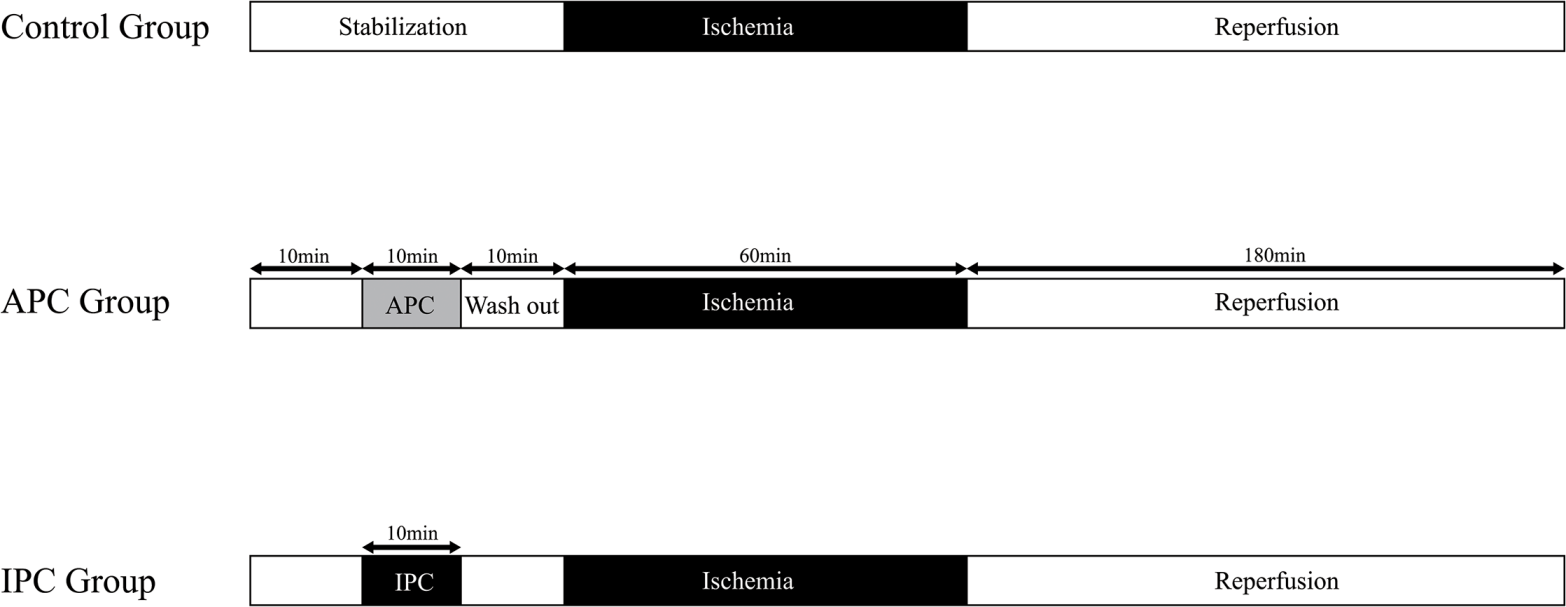
Diagram of the experimental protocol. Black areas represent the periods of hepatic ischemia. All animals were subjected to 60 min of ischemia and 180 min of reperfusion. A minimal alveolar dose of sevoflurane was administered for 10 min followed by a 10 min washout period in the APC group.APC, anaesthetic preconditioning; IPC, ischemic preconditioning

After 30 min of stabilization following surgery, rats were randomly divided into three groups (Fig 1): 1. Control group (mock preconditioning) (n = 7) rats were subjected to 60 min of hepatic ischemia followed by 3 h of reperfusion (I/R period was similar in all other groups); 2. APC group (n = 7) rats received sevoflurane (Maruishi Pharmaceutical, Osaka, Japan) at a volume of 2% (corresponding to one minimal alveolar concentration) for 10 min with a subsequent 10-min washout period prior to the I/R regimen; 3. IPC group (n = 7) rats were subjected to 10 min of hepatic ischemia followed by 10 min of reperfusion prior to ischemia.

Rats from each group were sacrificed at the end of the 3 h reperfusion period. Blood was sampled for measurement of the liver enzymes aspartate aminotransferase (AST) and alanine aminotransferase (ALT), and liver samples from the left lateral and median hepatic lobes (segments II–IV) were harvested within 3 min of sacrifice under terminal anaesthesia for miRNA measurement. Liver samples were washed twice with cold phosphate-buffered saline and immediately stored at −20°C in RNAlater solution (Applied Biosystems, Foster City, CA, USA). After 1 day, the RNAlater solution was rapidly separated from the samples by centrifugation (10,000 ×*g*, 4°C, 5 min). Total RNA was extracted using a mirVana miRNA Isolation Kit (Applied Biosystems). The RNA quantity and quality were assessed using a NanoDrop ND-1000 (Thermo Fisher Scientific, Waltham, MA, USA). A 260/280 nm reading of 1.8 or more was considered acceptable for quantitative analysis. The total RNA sample containing miRNAs was used for qRT-PCR.

### Serum transaminase assay

AST and ALT activities were measured in a clinical laboratory using CicaLiquid AST/ALT (KANTO KAGAKU Tokyo Japan) to assess hepatic function/damage, and the results are reported in units per litre.

### miRNA screening test

To determine the potential involvement of miRNAs in hepatic APC and IPC, comprehensive miRNA screening experiments were performed to determine miRNA levels in the rat liver after *in vivo* I/R. The miRNA expression profiles were assessed using the Megaplex qRT-PCR Low Density MicroRNA Array (TaqMan Low Density Array (TLDA); Applied Biosystems) under identical conditions. All procedures were performed as previously described.[18] In brief, for miRNA cDNA synthesis, RNA was reverse transcribed using a miRNA reverse transcription kit and the Megaplex RT Primer Pool A and B, and the product was mixed with TaqMan Universal PCR Master Mix (Applied Biosystems). qRT-PCR of 384 miRNAs was performed with the TaqMan MicroRNA Array using a 7900HT Fast Real-Time PCR System (Applied Biosystems).[19] Comparative Ct (number of cycles required for the fluorescent signal to cross the threshold, 2−ΔΔCt) analysis was performed to identify differentially expressed miRNAs following sevoflurane or partial ischemia exposure. The mammalian U87 small nuclear RNA was used for data normalization across the experiments.[20] Data were processed and exported using SDS v2.4 software (Applied Biosystems) with baseline settings and a threshold of 0.2, and were subsequently analysed using DataAssist software (Applied Biosystems).

### Pathway analysis

Gene networks and canonical pathways representing key genes were identified with Ingenuity Pathway Analysis (IPA; Qiagen, Redwood City, CA, USA; www.qiagen.com/ingenuity). IPA creates gene networks using the Ingenuity Knowledge Base, a database based on molecular interactions and gene-to-phenotype studies from over 200,000 peer-reviewed scientific articles. The networks include genes with direct and/or indirect interactions. For the direct gene relationships, the protein products have direct physical interactions with one another, such as enzymatic interactions. For the indirect gene relationships, the protein products do not physically interact, but their expression levels can be affected by each other. IPA can also identify biological functions that are statistically associated with certain genes belonging to a particular network. The data sets containing gene identifiers and corresponding fold changes and *P* values were uploaded into the web-based application, and each gene identifier was mapped to its corresponding gene object using IPA software.

### Statistical analysis

All values are expressed as means ± standard deviation. Only ≥ 2.0-fold changes in miRNAs were included for further analysis. Analysis of variance (ANOVA) followed by Tukey’s test (SPSS Statistics 21; IBM Corporation, Armonk, NY, USA) was used for comparison among the three groups. A p value of < 0.05 (two-tailed) was considered to be significant. IPA was used to calculate the significance value of a given pathway or network as the probability that the pathway or network was associated with the data set by random chance.

## Results

Successful ischemia after portal vein and hepatic artery occlusion for 60 min was confirmed visually (cyanosis) and by increases in AST and ALT levels. All rats survived until sacrifice, and the data for all rats were included in the analyses.

### Haemodynamic parameters

Although the blood pressure of APC group rats was lower than the other groups immediately after 2% sevoflurane treatment, none of the haemodynamic parameters differed among the groups throughout the study. We found no significant intra- or inter-group differences in heart rate or mean arterial blood pressure over time (Table 1).

**Table 1:** Systemic haemodynamic parameters.

	Baseline	Reperfusion0h	Reperfusion3h
Heart Rate (beats min ^-1^)			
Control	360.0 ± 30.0	328.6 ± 37.6	331.4 ± 73.7
APC	375.0 ± 34.7	335.7 ± 45.8	352.7 ± 50.8
IPC	346.7 ± 28.1	319.3 ± 31.4	362.0 ± 21.3
Mean arterial pressure (mm Hg)			
Control	107.8 ± 8.9	101.8 ± 4.2	94.7 ± 11.0
APC	110.8 ± 3.7	92.8 ± 19.0	102.1 ± 12.4
IPC	105.8 ± 5.0	95.9 ± 16.4	100.3 ± 17.0

Data are presented as means±SD.

APC, anaesthetic preconditioning; IPC, ischaemic preconditioning

### Serum transaminase levels

**Fig 2:**
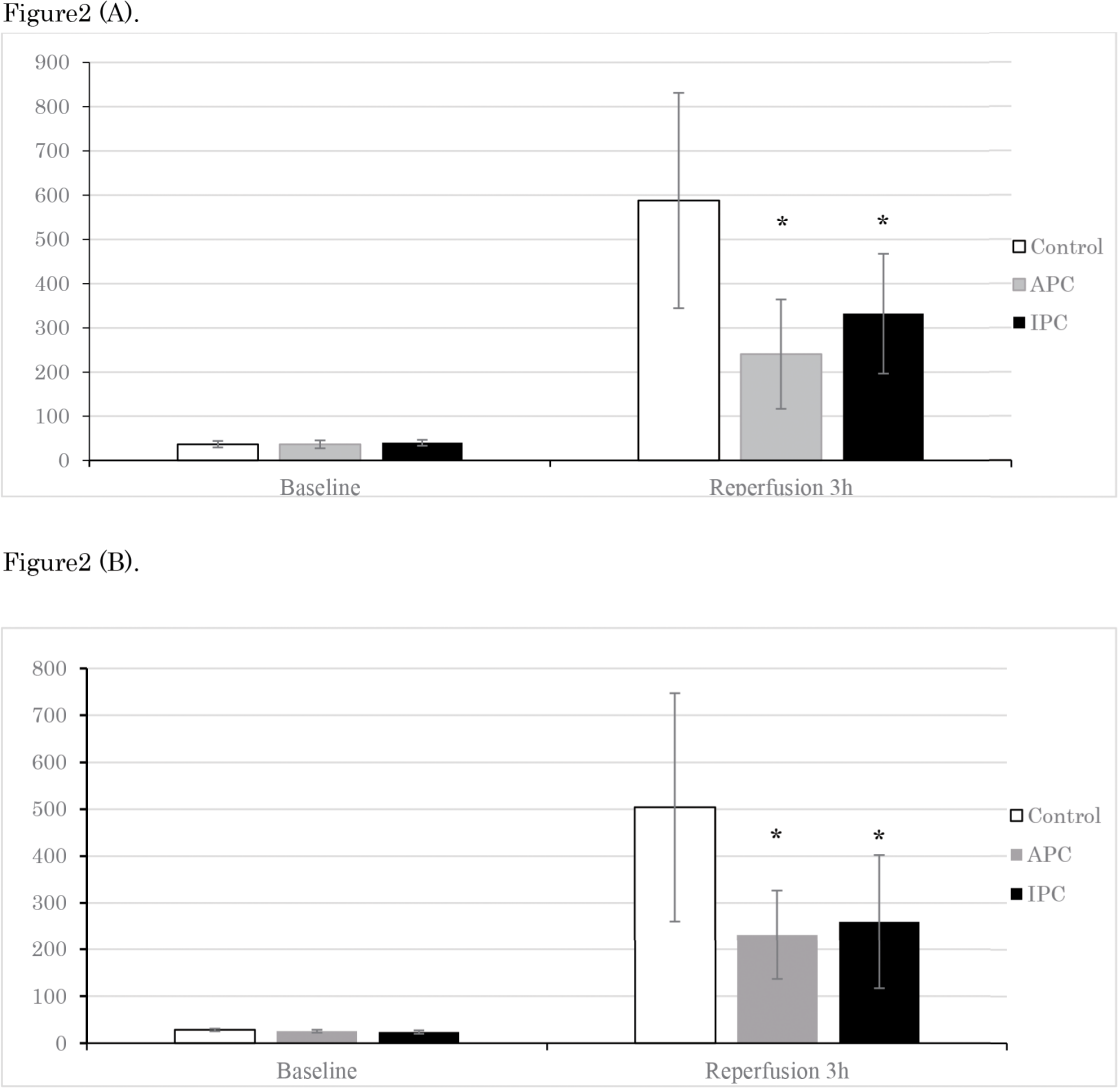
Serum levels of (A) aspartate aminotransferase (AST) and (B) alanine aminotransferase (ALT). The results are expressed as means ± SD. * p < 0.05 versus the control group.White: control group, grey: APC group, black: IPC group

The serum levels of ALT and AST, which are markers of hepatocyte injury, were significantly higher in the control group (ALT/AST: 503.7/587.7 units per litre) than in the APC (ALT/AST: 231.3 /240.4 units per litre, p < 0.05) and IPC groups (ALT/AST: 259.5/332.0 units per litre, p < 0.05). We found no significant difference in serum ALT or AST levels 3 h after hepatic ischemia between the APC and IPA groups (Fig 2A and 2B).

### Gene expression changes

Compared with the control group, up-regulation of three genes (2% of all detected genes) and down-regulation of 114 genes (98%) was observed in the APC group, whereas the up-regulation of three genes (1% of all detected genes) and down-regulation of 205 genes (99%) was observed in the IPC group. TLDA showed that 112 miRNAs (95%) altered in the APC group were also altered in the IPC group, with only a few miRNAs being significantly different between the two conditions.

### Pathway analysis

To identify genes selectively altered by APC and IPC, 213 genes that were either up- or down-regulated more than 2-fold compared with the control group were selected for further analysis. These genes were subjected to IPA to identify biological functions, pathways, and gene networks that were significantly affected by APC and IPC of I/R injury. Although these functions included inflammatory response and connective tissue disorders, only a few pathways have been reported to have a relation with ischemia reperfusion. We further focused on pathways indicated to have a relation with ischemia reperfusion, and miRNAs directly or indirectly related to these pathways.

**Fig 3:**
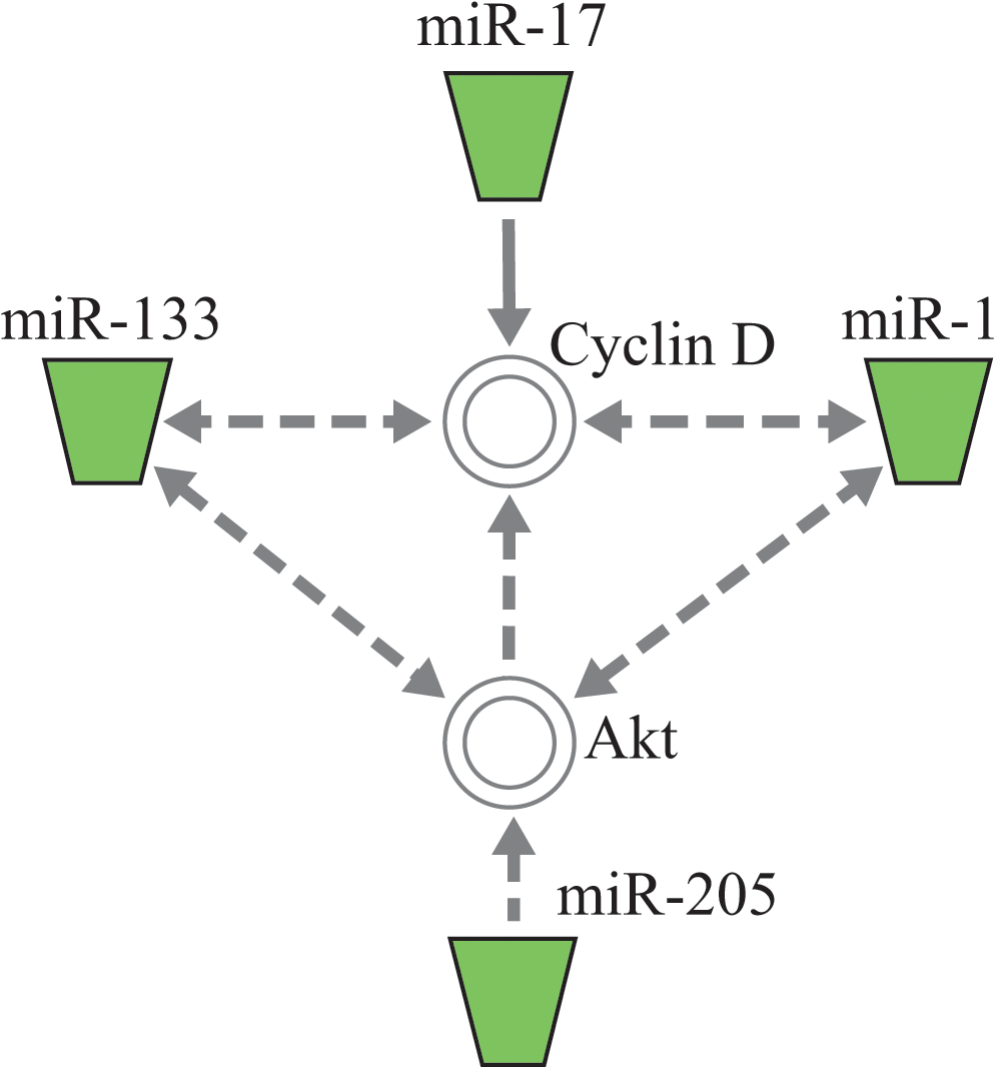
Functional network of down-regulated (green) microRNAs in the APC and IPC groups. All the miRs, except miR-17, repress Akt activation. miR-1, miR-17, and miR-133 suppress cyclin D expression. The image was created using the Ingenuity Pathway Analysis software. Solid lines represent direct interactions, and dashed lines represent indirect interactions.APC, anaesthetic preconditioning; IPC, ischemic preconditioning

Four miRNAs (miR-1, miR-17, miR-133, and miR-205) related to the Akt–GSK–cyclin D1 pathway were significantly down regulated by both APC and IPC treatment (*p* < 0.05, Table 2). All miRNAs except miR-17 repress Akt activation, and miR-1 and miR-133 indirectly suppress cyclin D1 expression. Only miR-17 directly suppresses cyclin D1 expression (Fig 3).

**Table 2:** Differentially expressed miRs related to the Akt-GSK-cyclin D1 pathway in the APC and IPC groups.

microRNA	APC		IPC	
	FC	p value	FC	p value
miR-1	-8.51	<0.01	-6.5	<0.01
miR-17	-6.34	<0.01	-8.83	<0.01
miR-133	-32.34	<0.01	-7.34	<0.01
miR-205	-37.26	<0.01	-1.96	<0.01

Data are presented as means±SD.

APC, anaesthetic preconditioning; IPC, ischaemic preconditioning

## Discussion

Our study showed a number of important findings. First, although haemodynamic parameters did not differ among the three groups, the serum AST/ALT ratio was significantly higher in the control group than in the APC and IPC groups. This suggests that APC and IPC provided similar levels of liver protection under the same I/R conditions. Second, comprehensive miRNA screening showed that, compared with the control group, most differentially expressed miRNAs in the APC group were common to those identified in the IPC group, indicating similar changes in gene expression between these groups. Finally, IPA identified four miRNAs related to the Akt–GSK–cyclin D1 pathway that were significantly affected by both APC and IPC treatments.

A recent study of the IPA knowledge database described relationships between these miRNAs and the Akt–GSK–cyclin D1 pathway, reporting that miRNAs decreased by APC and IPC suppress the Akt–GSK–cyclin D1 pathway.

Yu and colleagues revealed that phosphorylated Akt, a downstream target of the phosphatidylinositol-3-kinase (PI3K)/Akt signalling pathway, is up-regulated by decreased miR-1.[21] Similarly, Huang and colleagues found that miR-133 represses the insulin-like growth factor 1 receptor, which is upstream of PI3K/Akt signalling at the post-transcriptional level, and negatively regulates the PI3K/Akt signalling pathway.[22] Iorio and colleagues demonstrated that miR-205 impairs activation of the Akt-mediated survival and proliferation pathway *in vitro*.[23] A previous study also suggested that attenuation of miR-1/miR-133 transcription leads to the up-regulation of their direct downstream target cyclin D1,[24] and the abundance of cyclin D1 and expression of miR-17 were inversely correlated.[25, 26]

Activation of the Akt–GSK-3β pathway and cyclin D1 plays a key role in liver I/R injury.[27] Inhibition of GSK-3β by activation of Akt is thought to reduce I/R injury due to its inhibitory effects on mPTP opening and activation of the hepatocellular proliferation factor cyclin D1.

Akt is a primary mediator of the downstream effects of PI3K, preserving mitochondrial integrity by phosphorylating molecules such as GSK-3β.[8] Indeed, activating the Akt-GSK-3β pathway reduces cellular apoptosis and protects hepatic cells from I/R injury.[27,28]

Cyclins are related to cell cycle regulation, and cyclin D1 controls liver cell proliferation. Cai and colleagues investigated cell proliferation using flow cytometry and cell cycle analysis and found that IPC promotes cyclin D1 expression and hepatocellular proliferation.[10, 29] Furthermore, these authors confirmed that IPC could promote cyclin D1 expression and hepatocellular proliferation during early I/R injury. Other studies have reported that Akt–GSK-3β and cyclin D1 belong to the same pathway.[30, 31]

In the present study, we found decreased levels of miRNAs that suppress the Akt–GSK–cyclin D1 pathway in the APC and IPC groups. A previous study has shown that, of the various miRNAs, the down-regulation of miR-1, miR-133, and miR-17 causes activation of Akt and cyclin D1.

Therefore, we hypothesize that APC and IPC suppressed these miRNAs and led to the activation of the Akt–GSK3β–cyclin D1 pathway, thereby eliciting the observed protective effects on the liver.

It is important to note that one miRNA can regulate several different mRNAs, and a single mRNA can be regulated by several different miRNAs. Therefore, miRNAs represent a complex genetic regulation mechanism that can modulate biological processes at different levels.[32] Because of the complexity of this mechanism, we cannot exclude the possibility that other miRNAs and mRNAs may be involved in the Akt–GSK–cyclin D1 pathway.

In summary, our data demonstrate that APC and IPC induced changes in the expression of several common miRNAs, and IPA showed that these changes can alter the Akt–GSK–cyclin D1 pathway. Collectively, we conclude that APC and IPC are related to miR-1, miR-17, miR-133, and miR-205, which suppress the Akt–GSK–cyclin D1 pathway. This mechanism plays an important role in the protective effects of APC and IPC, but further studies are warranted.

## Supporting Information

S1 Fig Serum levels of aspartate aminotransferase (AST) and alanine aminotransferase (ALT). The results are reported in units per litre. APC, anaesthetic preconditioning; IPC, ischemic preconditioning
(XLSX)

S1 Table Systemic haemodynamic parameters. Heart Rate (beats min -1), Bloood pressure (mm Hg) APC, anaesthetic preconditioning; IPC, ischemic preconditioning. mBP, mean Blood Pressure. sBP, systolic Blood Pressure. dBP, diastolic Blood Pressure. HR, heart rate.
(XLSX)
